# Structure and function of gene regulatory networks associated with worker sterility in honeybees

**DOI:** 10.1002/ece3.1997

**Published:** 2016-02-16

**Authors:** Julia A. Sobotka, Mark Daley, Sriram Chandrasekaran, Benjamin D. Rubin, Graham J. Thompson

**Affiliations:** ^1^ Biology DepartmentWestern University1151 Richmond StreetLondonONN6A 5B7Canada; ^2^ Harvard Society of FellowsFaculty of Arts and SciencesHarvard University78 Mount Auburn StreetCambridgeMassachusetts; ^3^ The Broad Institute of MIT and Harvard415 Main StreetCambridgeMassachusetts

**Keywords:** Gene regulation, indirect selection, insect sociobiology, meta‐analysis, microarrays, RNA profiling, social behavior

## Abstract

A characteristic of eusocial bees is a reproductive division of labor in which one or a few queens monopolize reproduction, while her worker daughters take on reproductively altruistic roles within the colony. The evolution of worker reproductive altruism involves indirect selection for the coordinated expression of genes that regulate personal reproduction, but evidence for this type of selection remains elusive. In this study, we tested whether genes coexpressed under queen‐induced worker sterility show evidence of adaptive organization within a model brain transcriptional regulatory network (TRN). If so, this structured pattern would imply that indirect selection on nonreproductive workers has influenced the functional organization of genes within the network, specifically to regulate the expression of sterility. We found that literature‐curated sets of candidate genes for sterility, ranging in size from 18 to 267, show strong evidence of clustering within the three‐dimensional space of the TRN. This finding suggests that our candidate sets of genes for sterility form functional modules within the living bee brain's TRN. Moreover, these same gene sets colocate to a single, albeit large, region of the TRN's topology. This spatially organized and convergent pattern contrasts with a null expectation for functionally unrelated genes to be haphazardly distributed throughout the network. Our meta‐genomic analysis therefore provides first evidence for a truly “social transcriptome” that may regulate the conditional expression of honeybee worker sterility.

## Introduction

Eusocial breeding systems are characterized by a division of labor between reproductive and nonreproductive task specialists (Hölldobler and Wilson 2009). Eusociality is curious because any specialization toward a nonreproductive caste would seem unlikely to evolve. Yet, nonreproductive helper castes have evolved – and not just once, but on multiple occasions across the tree of life (Choe and Crespi 1997). Despite this apparent paradox, it is understood from inclusive fitness theory that even behavior costly to an individual's direct fitness can evolve, provided that the genes “for” that behavior are passed on through genetically related beneficiaries (Hamilton 1964; reviewed in Bourke 2011). Hamilton's theory of inclusive fitness explains just how, and under what conditions, the hypothetical genes for altruistic helping can evolve.

**Figure 1:**
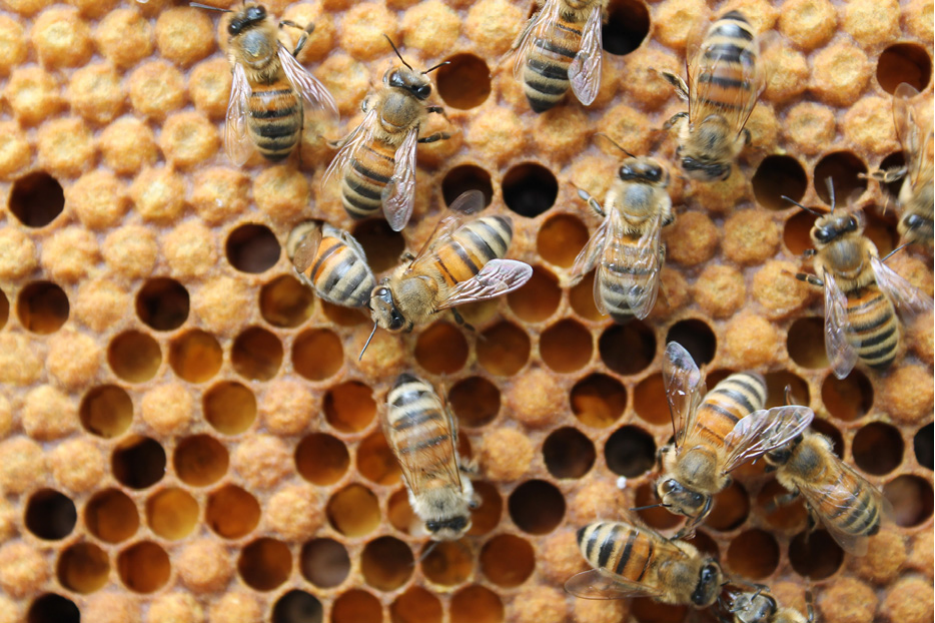
Workers on pupae and pollen. In the presence of their queen mother, members of this all‐female caste deactivate their ovaries and adopt alloparental roles within the colony. In the queenright condition, workers are essentially sterile. Kin theory predicts that “genes for sterility” evolve via reproducing relatives who carry, but clearly do not express, these genes. (Photograph: Emma K Mullen, Cornell University).

The honeybee *Apis mellifera* (Fig. 1) was the first eusocial animal to have its draft genome assembled (Weinstock et al. 2006), and as such has emerged as a preeminent model for social gene discovery (e.g., Zayed and Robinson 2012; Jasper et al. 2015; Mullen and Thompson 2015). Early screens have yielded hundreds of genes associated in their transmission or their expression with honeybee social traits, including genes associated with queen–worker caste differentiation (Evans and Wheeler 2001; Barchuk et al. 2007; Vojvodic et al. 2015), worker self‐sacrifice (Alaux et al. 2009), and even worker sterility (Oxley et al. 2008). This latter trait is especially interesting to behavioral genetics because it is so clearly nonreproductive, and as an example of reproductive altruism can only evolve via indirect selection, as predicted from Hamilton's rule. Genes for honeybee worker sterility have therefore become important to our understanding of how selection works via indirect fitness effects (Linksvayer 2015). Their identity provides a starting point to understand how reproductive altruism can evolve at the gene level.

To date, several studies have successfully screened the honeybee genome for genes associated with the conditional deactivation of worker ovaries and the functional expression of worker sterility (Grozinger et al. 2003, 2007; Thompson et al. 2006, 2008; Kocher et al. 2010; Cardoen et al. 2011; Wang et al. 2012). Most progress has come from exploiting the natural division of labor within living honeybee colonies. In queenright colonies, the queen signals her presence and fecundity to her thousands of worker daughters via pheromones, which have the effect of rendering the workers behaviorally selfless and functionally sterile (Hoover et al. 2003). When no queen is present, a proportion of workers (up to 30%) will activate their ovaries and begin to lay eggs. The queen mandibular pheromone (QMP) is important in this regard and bee behavioral geneticists have begun to experimentally manipulate the presence or absence of QMP to generate cohorts of workers with or without active ovaries (reviewed in Backx et al. 2012). From here, it is possible to screen for genes differentially expressed between effectively queenless (i.e., −QMP) workers with more active ovaries and queenright (i.e., +QMP) workers with deactivated ovaries. This type of assay can generate lists of genes for which changes in gene expression is functionally associated with worker sterility, but we do not yet know how these genes interact within multigene regulatory networks.

One opportunity to infer how genes for worker sterility are regulated in response to social cues is to examine their position and interrelationships within the honeybee transcriptional regulatory network (TRN). Chandrasekaran et al. (2011) have constructed a model of the honeybee brain TRN that describes the functional relationships between transcription factors (*n* = 380) and their target genes (*n* = 2002). This model provides an ideal scaffold upon which we can map lists of genes for sterility and plot their interrelationships. If these pheromone‐responsive genes evolved to coordinate the conditional expression of worker sterility, then we expect them to cluster as functional modules within the TRN. Here, we test this social transcriptome hypothesis by measuring the extent to which candidate genes for sterility cluster within Chandrasekaran et al.'s (2011) model TRN. First, we partition the network's topology into functionally related multigene clusters that, if present, would putatively regulate the expression of major but unspecified honeybee phenotypes. We then plot compiled gene sets for worker sterility onto the TRN, and test if their distribution is biased toward certain clusters (as opposed to randomly distributed among them). If so, we interpret this pattern as evidence for a “social transcriptome” that has evolved under, or is maintained by, indirect selection for reproductive self‐sacrifice.

## Materials and Methods

### Compiling and standardizing a meta‐data set

To assemble a comprehensive list of candidate genes for worker sterility, we performed a bibliometric search to identify studies that used QMP to identify genes differentially expressed upon exposure to ovary deactivating pheromone across a microarray. Specifically, we identified comparable studies that (1) used QMP as the primary stimulus for ovary deactivation (2) reported normalized gene expression differences between putatively ovary‐active (QMP−) and ovary‐inactive (QMP+) worker bees, and (3) used standardized rearing conditions to control for genetic and environmental background. If studies met these three inclusion criteria, we considered them for further analysis (Table 1). Prior to mapping the sterility gene lists onto the TRN, we standardized probe names from the different microarray studies into the common nomenclature of the honeybee's Official Gene Set (OGS, v3.2; Munoz‐Torres et al. 2011).

**Table 1:** Summary of microarray studies that have identified differentially expressed genes (DEGs) associated with the functional expression of worker sterility. A subset of DEGs are present in the honeybee brain transcriptional regulatory network (TRN) of Chandrasekaran et al. (2011)

Study	Experimental design	Tissue type	Total number of DEGs	Number of DEGs present in TRN
Grozinger et al. (2003)	QMP‐treated versus untreated workers in cages	Brain	1607	267
Thompson et al. (2006)	Wild‐type versus anarchist workers in colonies	Brain	20	2
		Abdomen	20	1
Grozinger et al. (2007)	QMP‐treated versus untreated workers in cages	Brain	94	18
Thompson et al. (2008)	Wild‐type versus anarchist workers in colonies	Brain	7	0
		Abdomen	5	0
Cardoen et al. (2011)	Ovary‐active versus ovary‐inactive workers in colonies	Whole Body	1292	255

QMP, queen mandibular pheromone.

### Visualizing the honeybee transcription regulatory network

To reconstruct the model TRN as a graph we first converted the gene–gene interaction information from a list format (available online: http://price.systemsbiology.net/honeybee-transcriptional-regulatory-network) into an adjacency matrix. We then imported this matrix into the graphing software package GEPHI (v0.8.1; Bastian et al. 2009), which functions as a network visualization tool. For display purposes, we used the “force atlas 2” layout algorithm (Jacomy et al. 2014) in GEPHI to visually maximize the internode distance on screen. We also chose to scale network nodes as a function of their degree – that is, the number of connections, such that the largest nodes on our graph reflect genes with the largest number of connections. These graphing options do not affect the underlying gene‐interaction matrix, but do help to view the complex network and to identify key genes and structural features that might not otherwise be apparent.

### Testing structure and function of the network

To test for evidence of functional clustering of coregulated genes within the total TRN, we partitioned the network's matrix into *k*‐groups of strongly interconnected genes. For this analysis, we used two algorithms: the Louvain clustering algorithm (Blondel et al. 2008) implemented in GEPHI and the GLay algorithm (Su et al. 2010) implemented in CYTOSCAPE (v2.8.3; Lopes et al. 2010). In each case, we allowed *k* to vary and chose the best‐fit model (value of *k*) that maximized the ratio of within‐ to between‐cluster connections, as measured by a *Q*‐score.

To test if our compiled gene sets for sterility map to specific clusters, we simply plotted them individually onto the TRN's topology and observed their distribution across clusters. In addition to mapping differentially expressed gene sets associated with sterility from microarrays, we mapped one alternate set of genes – the “hub genes” for worker sterility identified by Mullen et al. (2014). This set of *n* = 18 genes is likewise important to the social regulation of worker sterility, but they are not derived directly from a microarray. Rather, this latter gene set is already inferred to be tightly connected within networks, albeit cocitation networks that are derived independently of the present study.

We reason that if the coregulation of any of the above gene sets is more efficient than expected by chance, then they will be tightly interconnected within the TRN. Alternatively, if the coregulation of any candidate gene sets is coregulated inefficiently or not coregulated, then we do not expect them to cluster, and instead these genes should be haphazardly dispersed across the TRN's topology. We test this social transcriptome hypothesis in two ways. First, we use hypergeometric tests to compare the categorical distribution of genes among *k*‐groups of the TRN against the distribution expected under a null (haphazard) scenario. Second, we estimate the exact probability of observing the “maximum number of sterility genes” for any single group via randomization test, whereby we computationally shuffle gene labels across the TRN's topology (10^4^‐times) to generate a null probability of sterility genes per cluster.

As a final and independent test of adaptive efficiency in the coregulation of sterility genes, we test for biased use of c*is*‐regulatory motifs. For this analysis, we obtained a list of *cis*‐regulatory motifs in the 5KBp upstream region of each gene in the honeybee genome (described in Chandrasekaran et al. 2011). Briefly, motifs were identified using the swan program and statistically significant motifs were then associated with each gene (*P*‐value < 0.01). From the resultant gene‐by‐motif matrix (*n* = 603 unique motifs, available as Appendix S1) we tested if sterility gene sets were enriched for certain motifs, relative to expectation from their frequency in the honeybee genome as a whole. If so, we interpret this pattern as further evidence that the honeybee's transcriptome has been selected to regulate the conditional expression of worker sterility. For each gene set, we used a randomization test (based on 999 random samples of genes from the source) to generate *P*‐values for a chi‐square (*χ* ^2^) statistic. Expected gene counts for each *cis*‐regulatory motif were based on the null hypothesis that association with genes in the sterility gene set was proportional to their genomewide frequencies. When the distribution of a gene set among motifs differed significantly from expected (*α *= 0.05), we identified specific enriched motifs as those for which (1) the observed gene count (*O*) exceeded the expected (*E*) and (2) the normalized squared deviation ((*O* – *E*)^2^/*E*) was significantly greater than expected by randomization test (based on comparison with the maximum value of (*O*–*E*)^2^/*E* in the random samples).

### Gene set enrichment analysis

For any cluster of the TRN that appeared to be enriched for sterility genes, we estimated the biological functions of that cluster by performing a Gene Ontology (GO) analysis, as implemented in the online Database for Annotation, Visualization and Integrated Discovery (DAVID v6.7; Huang et al. 2009). Because DAVID is calibrated to certain model taxa (not including the honeybee, yet), we first converted our bee genes into *Drosophila melanogaster* homologues. Here, we assigned each translated bee gene sequence to its “reciprocal best hit” (Ward and Moreno‐Hagelsieb 2014) as performed using NCBI's BLASTp algorithm (v5.10). Any bee sequence without a clear one‐to‐one match (similarity score of *E*‐value < 10^−5^) was simply excluded from this part of the analysis. We used this bee‐to‐fly gene list to estimate the number of functionally related gene clusters, and retrieve any enriched GO annotation terms in DAVID (under default settings).

## Results

We assembled and normalized a unique set of candidate genes for sterility. This meta‐data set included genes from five published gene microarray studies (Table 1) and one gene network study (Mullen et al. 2014). In total, this meta‐data set identifies *n* = 4565 genes, as compiled from 10 different microarray experiments and nine cocitation networks. Not all genes from the source studies are modeled into the honeybee brain TRN. As a consequence, the meta‐data set captures approximately 12% of genes (*n* = 558 of 4565, available as Appendix S2) previously implicated in sterility. This 699‐gene set represents the total number of unique transcription factors and their predicted targets in the honeybee brain. These genes are highly relevant to the study of sterility and its underlying regulatory network.

**Figure 2:**
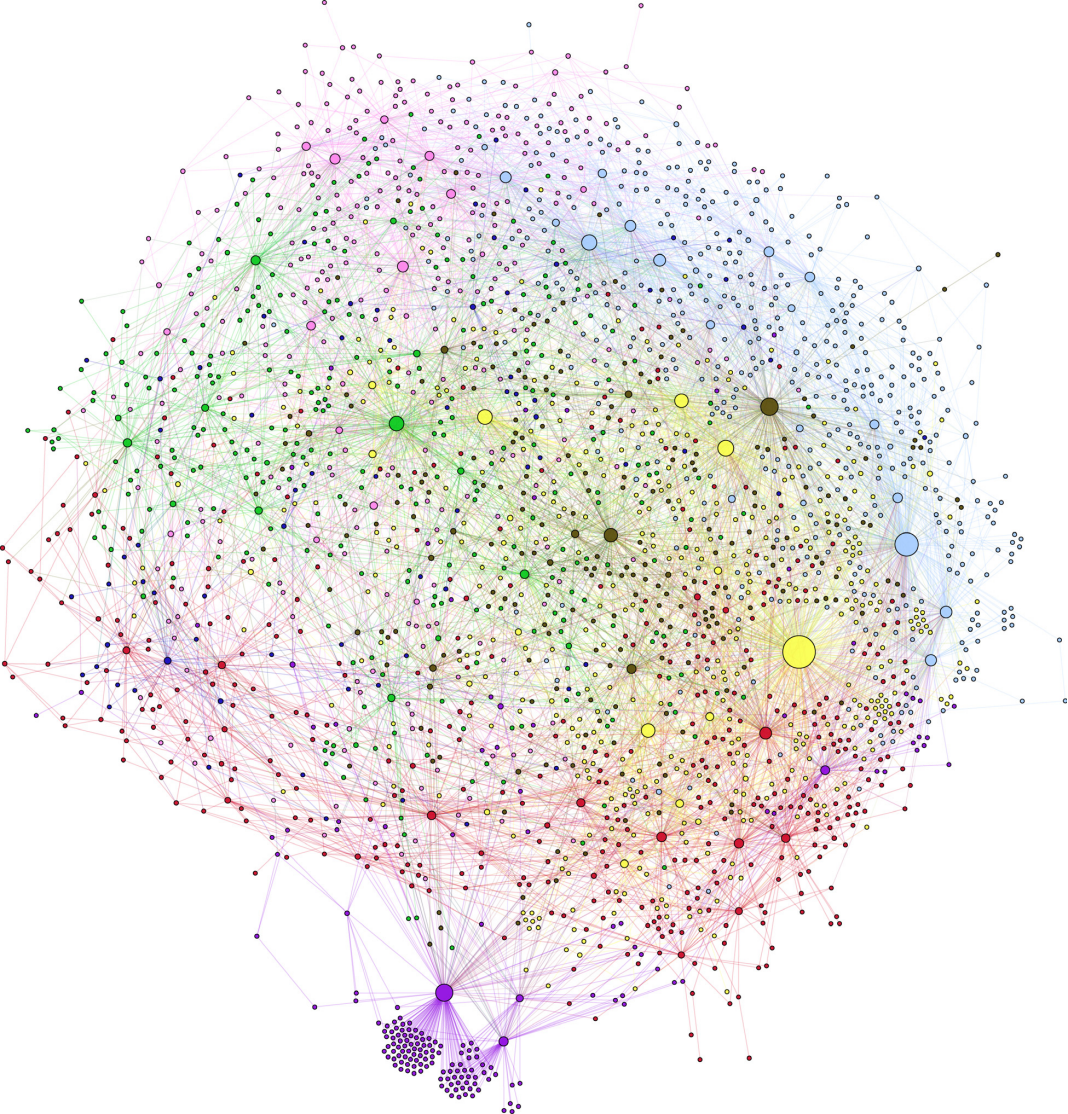
The reconstructed honeybee transcriptional regulatory network (TRN). The network contains 2382 nodes representing transcription factors and their putative target genes, and 6757 edges representing regulatory interactions. Colors denote the eight functional clusters that we infer through model fitting.

Our reconstruction of Chandrasekaran et al.'s (2011) TRN is shown in Figure 2. Overall the topology is highly structured. That is, the topology is not a random constellation of vertices (genes) and edges (connections), but rather shows well‐defined and visible clusters of genes that are densely packed and highly interconnected. At the single‐gene level, hub genes that control the regulation of other genes are evident by the large number of edges connecting them to their targets. By contrast, there are many weakly connected genes that are characterized by only a few functional connections. The number of edges connecting genes within the TRN varies from *n* = 1 (a gene with a single connection) to the most highly connected gene (*lag1*) with *n* = 393 connections to other genes. Moreover, cluster analysis suggests that the TRN consists of *k *=* *8 functional clusters. This value of *k* yielded a *Q*‐score of approximately 0.49 (*Q*‐score = 0.498 by Louvain clustering algorithm; *Q*‐score = 0.494 by GLay algorithm) and provides the best fit among the range of alternatives that we tested (*k *=* *1–27; the maximum number of clusters detected was 27). A major finding of our cluster analysis is therefore that the entire honeybee brain TRN may consist of as few as eight functional modules of highly interconnected genes.

**Figure 3:**
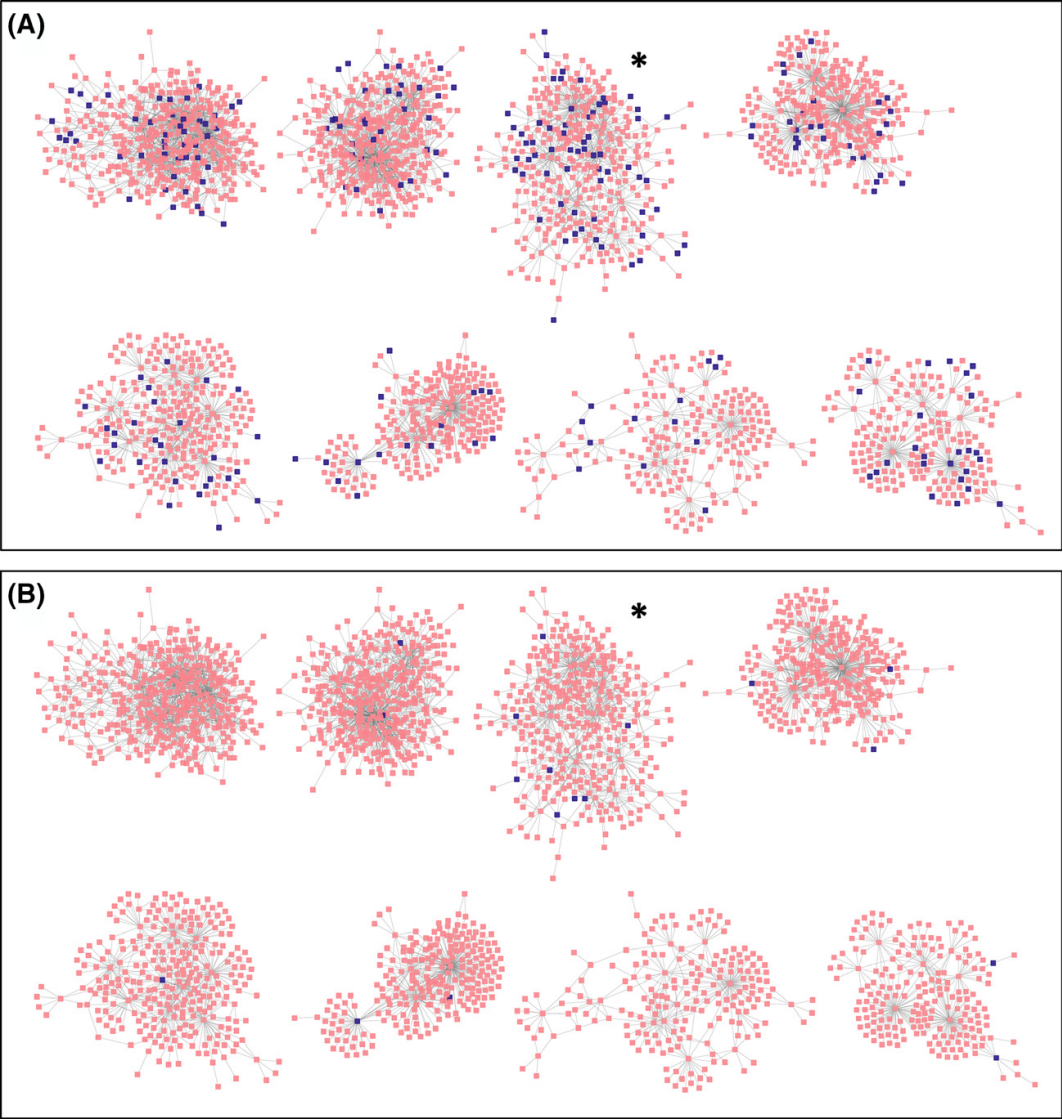
Sterility genes mapped onto the eight clusters that best describe substructure of the honeybee brain transcriptional regulatory network. The clusters are arranged from largest to smallest: Cluster 1, 433 genes; Cluster 2, 384 genes; Cluster 3, 361 genes; Cluster 4, 291 genes; Cluster 5, 281 genes; Cluster 6, 234 genes; Cluster 7, 199 genes; Cluster 8, 197 genes (Gene lists associated with each cluster are available as Appendix S3). For demonstration purposes we show “sterility genes” mapped as blue nodes from (A) Cardoen et al. (2011; *n* = 267 genes) and (B) Mullen et al. (2014, *n* = 18 genes). Of the four gene sets tested, these two represent the largest and smallest that are statistically biased toward Cluster 3, which is shown by the * symbol.

Figure 3 shows the TRN decomposed into its eight clusters, as displayed with different sets of sterility genes mapped by study‐of‐origin. The sterility gene lists from Thompson et al. (2006, 2008) had little representation on the TRN to statistically test their distribution across the eight clusters (*n* = 2 and 0 genes, respectively). The gene sets identified from the microarray studies of Cardoen et al. (2011, *n* = 255), Grozinger et al. (2003, *n* = 267), and Grozinger et al. (2007, *n* = 18) are, however, sufficiently large to permit a test of biased distribution via contingency table analysis. Genes converted from the Cardoen et al. (2011) and the Grozinger et al. (2003) studies are significantly biased in their distribution over the TRN such that sterility‐related genes are overrepresented in certain clusters (*χ* ^2^ = 21.7, *P *<* *0.01 for Cardoen et al. 2011; *χ* ^2^ = 88.2, *P *<* *0.01 for Grozinger et al. 2003). Genes converted from Grozinger et al.'s (2007) study are, by contrast, not biased with respect to cluster (*χ* ^2^ = 11.4, *P *=* *0.13) and their distribution across clusters within the TRN appears haphazard. Finally, hub genes identified from the cocitation network analysis of Mullen et al. (2014; *n* = 18) are also biased in their distribution over clusters (*χ* ^2^ = 15.2, *P *=* *0.03).

All three of the sterility gene sets with a nonrandom distribution over the topology were overrepresented in one of the predefined clusters of the TRN. That is, the third‐largest cluster (“Cluster 3” in Fig. 3) is enriched for sterility genes from each of the Cardoen et al. (2011), Grozinger et al. (2003), and Mullen et al. (2014) studies. The Grozinger et al. (2007) study was not significantly biased to a single cluster but nonetheless more if its genes localized to this cluster than any other. This pattern, where genes sampled from separate empirical studies converge onto a single cluster of the TRN, is highly significant: The estimated probabilities of observing 63 (of 255) genes from the Cardoen et al. (2011) set, 88 (of 267) genes from the Grozinger et al. (2003) set, and fully 8 (of 18) from the Mullen et al. (2014) set in “Cluster 3” are *P *<* *0.002 in all cases. The probability of observing six (of 18) genes from Grozinger et al. (2007) is *P *≈* *0.239.

In total, there are *n* = 136 unique sterility genes (*n* = 26 transcription factors and *n* = 110 targets) that map to Cluster 3, compared to *n* = 351 genes that map to one of the other seven clusters. This cluster in particular therefore appears to be functionally associated with the regulatory control of worker ovary deactivation and with the conditional expression of sterility. This cluster of the TRN, consisting of 361 genes (220 of them with one‐to‐one orthologues in *D. melanogaster*, available as Appendix S4), is enriched for genes related to regulation of transcription, cell morphogenesis, behavior, and imaginal disk pattern formation and development, among other biological processes (Table 2).

**Table 2:** Gene‐term enrichment analysis for genes within “Cluster 3” of the honeybee transcriptional regulatory network, as inferred by comparison with “reciprocal best hit” homologues in *Drosophila melanogaster*. We here use DAVID's Functional Annotation Clustering tool to isolate the single most enriched term from each Annotation Cluster with an Enrichment Score > 1.5 (8 of 36)

Annotation cluster	Enrichment score	Biological process (GO ID)	Gene count	*P*‐value
1	2.92	Regulation of transcription (GO:0006355)	32	6.7E‐6
2	2.65	Cell morphogenesis (GO:0000902)	20	1.5E‐4
3	2.16	Behavior (GO:0007610)	19	3.0E‐4
4	1.76	Imaginal disk pattern formation (GO:0007447)	8	1.1E‐3
5	1.74	Wing disk development (GO:0035220)	14	8.9E‐4
6	1.72	Imaginal disk development (GO:0007444)	20	5.7E‐5
7	1.54	Cell–cell signaling (GO:0007267)	13	2.2E‐4
8	1.54	Open tracheal system development (GO:0007424)	9	4.3E‐3

The presence of common *cis*‐regulatory motifs also suggests a high level of functional coupling among the sterility genes. We identified statistically overrepresented *cis*‐regulatory motifs in the upstream region of sterility genes compared to the rest of the genome and found that sterility gene sets often shared evolutionarily conserved motifs. Specifically, we found that genes identified from the Cardoen et al. (2011; *χ* ^2^ = 546.94, *P *=* *0.013), Grozinger et al. (2003; *χ* ^2^ = 590.20, *P *=* *0.006), and Mullen et al. (2014; *χ* ^2^ = 506.28, *P *=* *0.033) studies share common upstream motifs. The single most gene‐loaded motifs are as follows: *bab1* (*n* = 108 genes; from gene set of Cardoen et al. 2011; *P *=* *0.040), *V_EGR1_01* (*n* = 44 genes; from gene set of Grozinger et al. 2003; *P *=* *0.002) and V_GRE_C (*n* = 5 genes; from gene set of Mullen et al. 2014; *P *=* *0.098).

## Discussion

According to the social transcriptome hypothesis, pheromone‐responsive genes evolved under indirect selection to coordinate the conditional expression of worker sterility, and therefore form functional modules within the honeybee transcriptional regulatory framework. Overall results support this hypothesis. First, we show that multiple sets of genes implicated in queen pheromone‐induced sterility tend to locate to a single predefined topological region of the honeybee brain TRN. This convergence of gene sets into a single module suggests that the meta‐data successfully capture an underlying regulatory signal related to the pheromone deactivation of worker ovaries – that is, “sterility”. Further evidence for functional coupling of sterility genes that is not dependent on the network is found in the statistical overrepresentation of particular *cis*‐regulatory motifs in the upstream regions of these genes. Together, our topology‐ and motif‐based analyses suggest that sterility genes have a greater likelihood of being influenced by the same set of transcription factors. We have therefore detected evidence for a functional module within the honeybee brain transcriptional network that putatively regulates worker sterility in response to social cues.

We do not know the extent that this module evolved under direct versus indirect selection – that is, for selfish versus selfless reproduction, or the extent that the module is used to regulate other behavioral reproductive functions in the honeybee worker caste (Chandrasekaran et al. 2011; Molodtsova et al. 2014). However, given that sterility in this context is a form of reproductive altruism, we make the reasonable assumption that the multigene module implicated here is at least partially evolved or maintained by indirect selection of the type specifically invoked by inclusive fitness theory. Our inference for selection on the regulation of this worker‐associated trait is consistent with recent population genomic studies by Harpur et al. (2014), Wallberg et al. (2014), and Vojvodic et al. (2015) who each report that genes associated in their expression with worker phenotypes (although not specifically sterility) evolve rapidly under selection, implying worker phenotypes are well adapted despite having essentially no direct fitness. To the extent that these worker‐associated adaptations are truly selfless, then the selection implied in these studies is indirect, too.

Our cluster analysis of Chandrasekaran et al.'s (2011) TRN reveals that the model may be composed of as few as eight interconnected subnetworks. The convergence onto a value of *k *=* *8 between two different unsupervised clustering algorithms suggests that this estimate is robust to minor differences in method and assumptions, and reveals an underlying architecture to the brain‐centered regulation of honeybee behavioral phenotypes. Sterility genes sets were not used to reconstruct the TRN, so the evidence for their colocation to a specific cluster is not likely the result of an inherent bias in the training data for the TRN. This surprising result suggests that the regulation of sterility genes is shared with other behavior processes that were used to construct the TRN, including socially important behaviors like worker aggression, worker maturation, and worker foraging (Chandrasekaran et al. 2011). This underlying architecture for the genetical regulation of worker behavior may prove useful for identifying sets of genes that are associated with variation in the major types of worker bee behavior – for example, as might be relevant to apicultural performance.

We sourced our candidate gene lists from published studies that used microarray analysis of different tissue types, not just brain tissue. Cardoen et al. (2011), for example, use whole body tissue to detect genes differentially expressed as a function of pheromone mediated ovary deactivation, and still, these genes tend to cluster on the brain TRN. This convergence of “whole body genes” to the same location as “brain genes” suggests that the core structure of the honeybee regulome is conserved (Molodtsova et al. 2014) and that honeybee workers may in fact use a single module to specifically regulate sterility from beginning to end – that is, from the initial perception of queen pheromone in the brain and antennal regions of individual workers to the downstream deactivation of ovaries in their abdomens. Future functional genomic studies that attempt to perturb the module will provide an important test of this hypothesis.

Cluster 3 in particular is implicated in the specific regulation of worker sterility – a complex phenotype that, at the proximate level, is underlain by the perception of queen signal and the suppression of personal reproduction among workers via ovary deactivation (Hoover et al. 2003; Backx et al. 2012). It is understood that the efficacy of queen pheromone to suppressing reproduction is very high among the worker population (only ~0.1% eggs in a queenright colony are worker‐laid; Visscher 1989). Under queenless conditions, however, only a minor proportion of workers may activate their ovaries to lay unfertilized eggs. Despite this “noise” in response to presence/absence of QMP, the published studies from which we sourced our meta‐data appear to reflect clear gene expression patterns associated with QMP‐induced sterility, as reported in each original study and in one prior meta‐analysis (Mullen et al. 2014). There is one gene set examined here, however, that showed only marginal evidence of clustering over the TRN, which may simply reflect the relatively small proportion of genes from Grozinger et al. (2007) that were actually present within the model TRN.

When coupled with alloparental care and colony defense, the functional sterility of honeybee workers is a short‐form example of reproductive altruism (Mullen and Thompson 2015) and, as such, is predicted to have evolved under indirect selection for coordinated gene expression that positively affect the fitness of nondescendant kin (Hamilton 1964; Bourke 2011; Thompson et al. 2013). Cluster 3 may therefore regulate the kin‐selected expression of sterility, and should now be targeted by functional genomic technologies (e.g., knockdown of hub genes) to verify, or reject, its role in mediating trade‐offs between direct (ovary‐active) and indirect (ovary‐inactive) fitness. To facilitate future knockdown studies we identified the top 20% most connected genes within Cluster 3 and nominally considered them as hubs. By this criterion, we identified four genes with more than 40 connections each (vs. fewer than eight connections for remaining 357 genes). The hub genes of Cluster 3 are as follows: *ftz‐f1, fru, GAGA‐like*, and *Dsp1*. Two of these are previously implicated in reproductive regulation. The *ftz‐f1* gene, with 145 connections, mediates a worker's response to juvenile hormone (Wang et al. 2012), a hormone that in itself regulates honeybee maturation, and is upregulated in ovary‐active bees (Cardoen et al. 2011). Likewise, the *fru* gene, with a total of 60 connections, is a male courtship regulator gene in *Drosophila* (Nilsson et al. 2000), and is downregulated in workers upon exposure to QMP (Grozinger et al. 2003). The *GAGA‐like* and *Dsp1* genes have 62 and 43 connections, respectively, and we here implicate them as targets for testing genetic effects on worker sterility. Overall Cluster 3 was enriched for several biological processes potentially related to the regulation of reproduction. Among them are imaginal disk development (GO: 0007444) and wing disk development (GO: 0035220), which, in ants – another eusocial taxon – are linked to caste differentiation and winglessness of the less reproductive caste (Abouheif 2004; Sameshima et al. 2004).

As a network‐independent test for the functional coupling of sterility genes, we found an overrepresentation of *cis*‐regulatory motifs in their upstream regions. Our null expectation was for proportional usage of motifs, based on their estimated frequency in the honeybee genome as a whole. Instead, we found that a subset of motifs is overrepresented in the promoters of sterility genes. This pattern suggests that there are a small number of transcription factors that regulate a relatively large number of sterility genes – an efficient mechanism that potentially evolved via expansion of a family of factors that share the same motif. Among these overrepresented motifs are *bab1*, which is associated with bric‐à‐brac nuclear proteins required for the proper development of ovaries (Couderc et al. 2002) and other tissues in *D. melanogaster* (Lours et al. 2003). We therefore implicate the *bric‐à‐brac* homologue in *A. mellifera* (LOC725189) as potentially responsive to social cues and important to the reproductive dimorphism between ovary‐active (reproductive) and ovary‐inactive (sterile) workers.

Finally, during standardization of the gene lists, a subset (1449 of 3221; 45%) of ESTs (from Grozinger et al. 2003, 2007 studies) did not match the OGS, and this subset of probes was therefore not included in our analysis. This exclusion simply reflects updates and ongoing corrections to OGS annotations (Elsik et al. 2014). Furthermore, the TRN only describes the interactions between predicted transcription factors and their putative target genes. As such, only a subset of sterility genes from each study map onto the TRN. The rest, derived from microarray studies of brain (Grozinger et al. 2003) or whole body (Cardoen et al. 2011) tissue, are presumably not part of the honeybee brain TRN, but may still be important to the ultimate expression of functional sterility. Nonetheless, our findings suggest that honeybee evolution has been characterized by selection for a social transcriptome, such that its features may be shaped in part by indirect fitness effects. This finding is significant to the field of insect sociobiology in two ways. First, indirect fitness effects must be important to the evolution of reproductive self‐sacrifice (Thompson et al. 2013; Linksvayer 2015; Vojvodic et al. 2015), yet molecular evidence for these effects remains rare. Second, the evidence for functional clustering among sterility genes in honeybee implied here stands in some contrast to the lack of evidence for any physical clustering of socially relevant genes into linkage groups on the honeybee genome, as they are in some species of (eusocial) ants (Purcell et al. 2014). Evidence for a “social transcriptome” may therefore help explain the emergence of social life in some insect genomes.

## Data Availability

Data files associated with this study are available as Supporting information.

## Conflict of Interest

None declared.

## Supporting information


**Appendix S1.** Honeybee *cis*‐motif data.

**Appendix S2.** List of sterility genes represented on the network.

**Appendix S3.** Gene lists associated with each network cluster.

**Appendix S4.** Cluster 3 genes with reciprocal best‐hit matches to *Drosophila*.
